# The complete mitochondrial genome of *Hericium erinaceus* (Bull.:Fr.) Pers., 1797 (Russulales, Basidiomycota): an edible and medicinal fungus

**DOI:** 10.1080/23802359.2024.2324923

**Published:** 2024-03-19

**Authors:** Mei Wang, Fei Xu, Xiaomei Hu, Jianfei Chen, Xiaoya Song, Tingting Song

**Affiliations:** a College of Life Science, Northeast Agricultural University, Harbin, People’s Republic of China; b Institute of Horticulture, Zhejiang Academy of Agricultural Sciences, Hangzhou, People’s Republic of China; c State Key Laboratory for Managing Biotic and Chemical Threats to the Quality and Safety of Agro-products, Zhejiang Academy of Agricultural Sciences, Hangzhou, People’s Republic of China; d Lishui Academy of Agricultural and Forestry Sciences, Lishui, People’s Republic of China

**Keywords:** *Hericium erinaceus*, mitochondrial genome, phylogenetic analysis

## Abstract

*Hericium erinaceus* (Bull.:Fr.) Pers., 1797, is an edible and medicinal fungus found in China. In this study, specimens of *H. erinaceus* HE0021 were collected from southeastern China (Yunhe County, Lishui City, Zhejiang Province, 28°7′12″N, 119°34′12″E). The whole mitochondrial genome of *H. erinaceus* HE0021 was sequenced using next-generation sequencing (NGS) technology, which comprised 15 protein-coding genes (PCGs), 27 transfer RNAs (tRNAs), two ribosomal RNAs, with a total length of 83,518 base pairs (bp). The results of the phylogenetic analysis show that *H. erinaceus* and *H. coralloides* were clustered in the same clade. The complete mitogenome sequence provides essential data for the subsequent investigation of *Hericium* and Russulales.

## 1. Introduction

The genus *Hericium* (within the classification Basidiomycota, Agaricomycetes, Russulales, and Hericiaceae) is a precious medicinal and nutritional resource. *Hericium* mycelium requires a moist, ventilated environment for growth, with a temperature ranging from 18 to 20 °C, and usually grows upon injured or recently fallen dead hardwood (mostly oak and pine). As a traditional edible and medicinal mushroom, *Hericium* is also called ‘delicacies from the mountains monkey head’ in China (Wang et al. 2009). Research has shown that the fruit body of *Hericium* is rich in protein, fat, polysaccharides, crude fiber, alkaloids, and sterols, among which polysaccharides and sterols were mainly functional compounds and got more attention. Medical research has demonstrated that it has anti-ulcer, anti-inflammation, anti-tumor, anti-aging, and liver protection effects; it is also reported in traditional Chinese medicine theory that its functional compounds possess the potential to control hyperglycemia, hypertension, and hyperlipidemia (Mizuno et al. 1992; Wang 2001; Lee et al. 2009). Four species have been identified in China, *Hericium erinaceus* (Bull.:Fr.) Pers., *Hericium caput-medusae* (Bull.:Fr.) Pers., *Hericium laciniatum* (Leers) Banker, and *Hericium coralloides* (Scop.:Fr.) Pers. ex Gray. Among them, *H. erinaceus*, the main species in the genus of *Hericium*, also known as saprotrophic fungi, has round and thick basidiocarps, white to off-white flesh, and is slightly translucent and rubbery. It is often found hanging onto the trunks of trees. The surface of the pileus is full of needle-like thorns after maturity. It is shaped like a monkey’s head, hence the Chinese name ‘Hou tou jun’ or ‘Ci wei jun’ (Wang et al. 2009). There is one *H. erinaceus* reference genome based on the strains CS-4 (Gong et al. 2020), and the mitochondrial sequence is still absent from the reference genome. Therefore, in this study, the complete mitochondrial sequence of *H. erinaceus* HE0021 was assembled and compared to other Agaricomycetes species. This result could be helpful for species identification and the genetics and evolutionary processes of *Hericium* and other Agaricomycetes species.

## 2. Materials and methods

### 2.1. Sample collection and preservation

**Figure 1.**
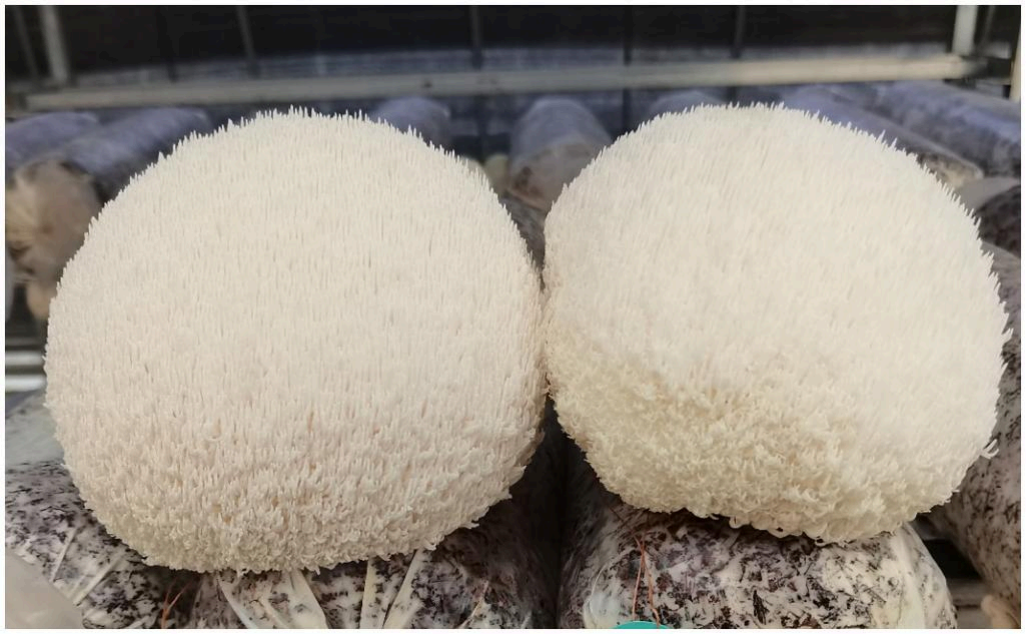
The fruit body of 2017-HE0021 strains (the photograph is courtesy of Xiaoya Song).

The voucher specimens of *H. erinaceus* were collected from a small county named Yunhe (28°7′12″N, 119°34′12″E), within Lishui City, Zhejiang province, on 14 March 2017. The specimens were deposited in the Herbarium, Institute of Horticulture, Zhejiang Academy of Agricultural Sciences, Hangzhou, People’s Republic of China (http://www.zaas.ac.cn/, TT, Song, songtingting@zaas.ac.cn) with the voucher number 2017-HE0021 (Figure 1).

### 2.2. DNA sequencing and cleaning of raw reads

The mycelium of HE0021 was isolated from the fruiting body, which is collected from dead wood, grown in a potato dextrose agar (w/v: 2% potato boiled for potato extract broth, 2% glucose, and 2% agar) (PDA) medium. It was identified as *H. erinaceus* based on its morphological characteristics (Wang et al. 2009) and ITS gene sequence blast result in NCBI (100% similar to *H. erinaceus* MT448853, Figure S1) by the Institute of Horticulture, Zhejiang Academy of Agricultural Sciences, Hangzhou, People’s Republic of China. The amplification of internal transcribed spacer (ITS) region of rDNA was carried out using universal primers ITS1 (5′-TCCGTAGGTGAACCTGCGG-3′) and ITS4 (5′-TCCTCCGCTTA TTGATATC-3′). Genomic DNA was extracted from the mycelium using a DNAsecure Plant Kit (Tiangen Biotech Co. Ltd., Beijing, China). An aliquot of purified DNA (0.2 μg) was then fragmented by sonication to a size of 350 bp. Subsequently, a short-insert (350 bp) library was constructed using the Nextera XT DNA library preparation kit (Illumina, San Diego, CA). The DNA libraries were sequenced on the Illumina NovaSeq 6000 platform, generating 150 bp paired-end reads. The raw data were edited using next-generation sequencing (NGS) QC Tool Kit v2.3.3 (Patel and Jain 2012). Finally, a total of 16,868,580 clean reads were obtained from the library of *H. erinaceus.*

### 2.3. Assembly, annotation, and visualization

High-quality sequence data (5.1 G) were selected for the assembly of the complete mitochondrial genome using CLC Assembly Cell Packages v4.2.1 (Qiagen, Aarhus, Denmark) with default parameters. The online MITOS server was used to annotate other genomic elements (Bernt et al. 2013), and the assembly process utilized the *H. coralloides* mitochondrial genome sequence as a reference (GenBank accession number: KY007042) after predicting the coding genes using fgenesB (Solovyev and Salamov 2011). All predicted genes were blasted against the NCBI nr nucleotide with 10^−5^ as the cut-off *e*-value to check their validity. A circular map of its complete mitochondrial genome was visualized using SnapGene (GSL Biotech LLC, San Diego, CA).

### 2.4. Phylogenetic reconstruction

We download 21 other mitogenome sequences from NCBI, including 21 species belonging to Russulales, the whole Lactarius/Russula clade as an outgroup to *Hericium*. The 15 common protein-coding genes (PCGs) (*atp6*, *atp8*, *atp9*, *cob*, *cox1*, *cox2*, *cox3*, *nad1*, *nad2*, *nad3*, *nad4*, *nad4l*, *nad5*, *nad6*, and *rps3*) in each complete mitochondrial genome of 21 species were aligned with the genes in *H. erinaceus* using MAFFT 7.490 (Katoh and Standley 2013) with the FFT-NS-2 strategy. Then the aligned genes were concatenated. Finally, Iqtree 2.0 was utilized to construct a phylogenetic tree with 1000 bootstraps based on the maximum-likelihood (ML) method (Nguyen et al. 2015). The built-in model-finder in Iqtree determined the best model to be GTR + F + I + G4 based on Bayesian’s criteria (Kalyaanamoorthy et al. 2017).

## 3. Results

**Figure 2.**
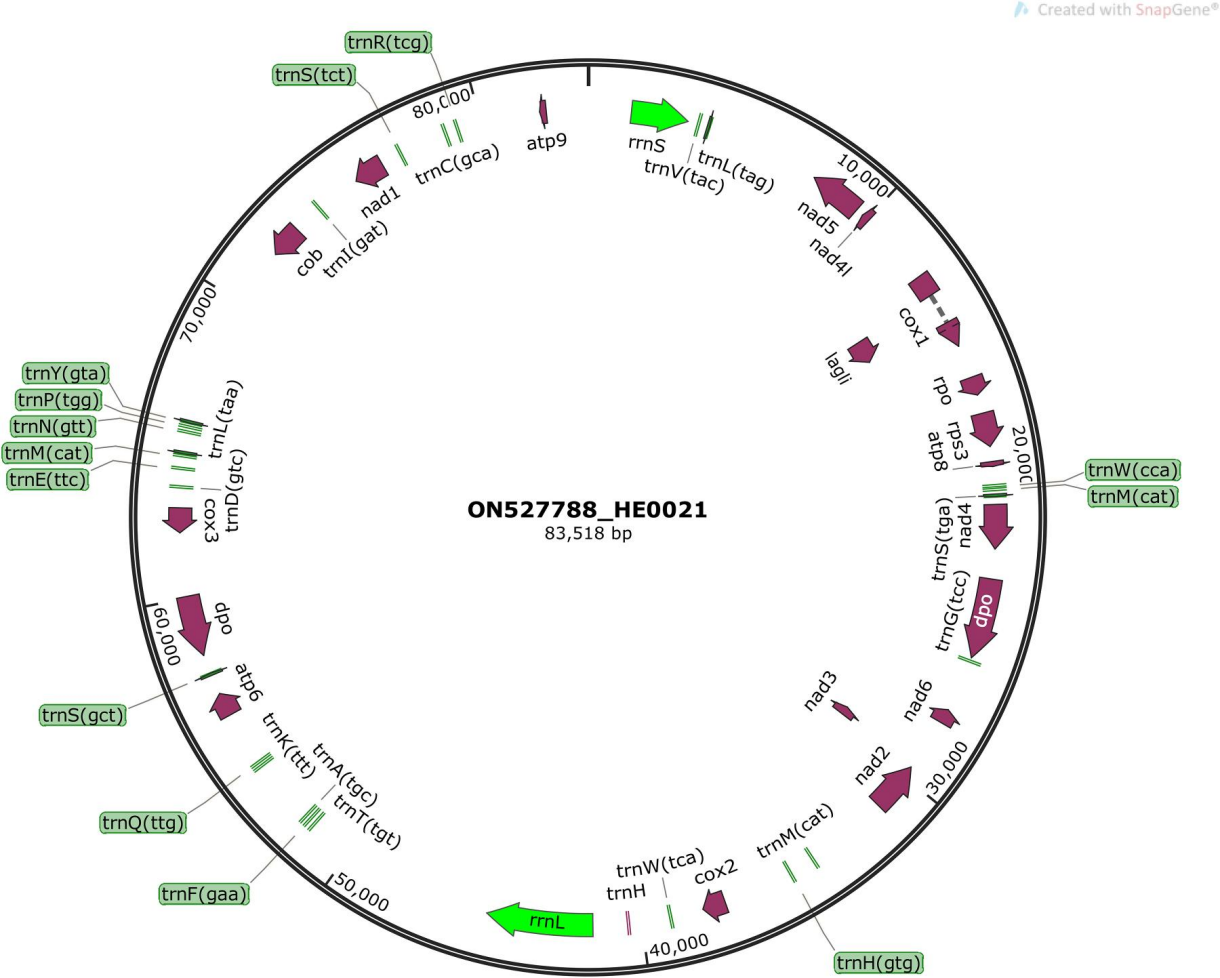
Schematic circular map of the assembled mitochondrial genome of *H. erinaceus* HE0021.

The average read mapping depth of the assembled mitochondrial genome was above ×7000 (Figure S2). The complete mitochondrial genome of *H. erinaceus* HE0021 is 83,518 base pairs long, possessing a double-stranded molecular weight of 51.579 MDa. The mitochondrial genome contains 49 genes consisting of 15 PCGs, 27 transfer RNA genes (tRNAs), and two ribosomal RNA genes (rRNAs) (Figure 2). The genome has an obviously biased A + T content of 81.7%, and the slightly positive AT-skew (0.006) and GC-skew (0.024). There is one PCG (*Cox1*) uses TTA as the start codon, and the rest of the 15 PCGs start with the ATG initiation codon. The lengths of tRNAs range from 69 to 86 bp. The *rrnS* and *rrnL* lengths are 1874 bp and 3488 bp, respectively.

**Figure 3.**
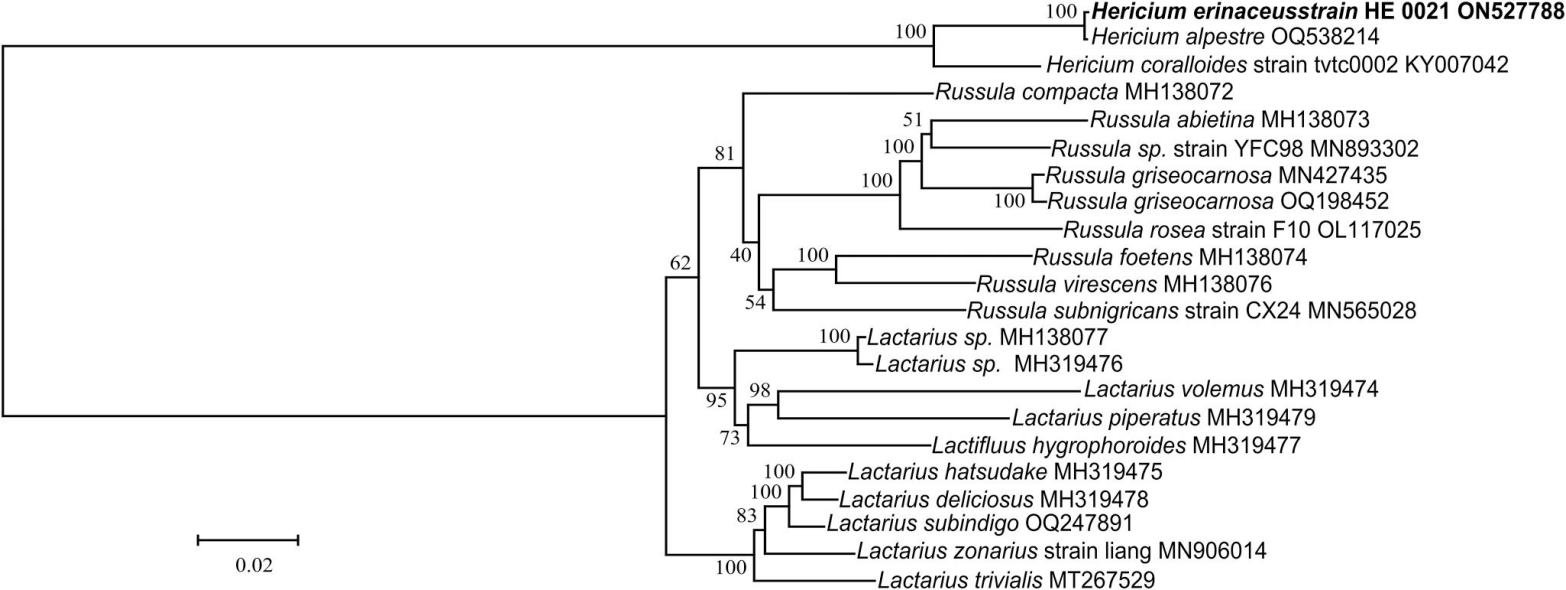
The maximum-likelihood (ML) phylogenetic position of *H. erinaceus* based on the 15 common protein-coding genes in each complete mitogenome sequence from 22 species. The values on the branches are the ML bootstrap percentages. The mitogenome of *H. erinaceus* determined in this study is marked by bold-type. Scale bar is for substitutions per site. The following sequences were used: *Russula subnigricans*, *R. virescens*, *R. abietina*, *R. compacta*, and *R. foetens* (Yu et al. 2019), *R. griseocarnosa*, *R. rosea*, *Lactarius deliciosus*, and *L. hatsudake* (Yu and Liang 2022), *L. trivialis* (Shao et al. 2020), *L. hygrophoroides* (Cai et al. 2021), *L. volemus* (Sun et al. 2020), *H. coralloides* (Zhang et al. 2017), and *H. alpestre*, *Russula sp.*, *L. zonarius*, *Lactarius sp.*, *L. subindigo*, and *L. piperatus* (unpublished).

The GTR + F + I + G4 model was used to reconstruct the ML tree. The phylogenetic analysis revealed that *H. erinaceus* and *H. coralloides* belong to the same clade, although discernible genetic diversity exists between the two species (Figure 3).

## 4. Discussion and conclusions

In this study, we reported the complete mitogenome sequence of *H. erinaceus* for the first time. The assembly circular mitochondrion was 83,518 bp in length. The phylogenetic analysis results indicate that *H. erinaceus* and *H. coralloides* are in one clade, but genetic diversity exists between the two species. The complete mitochondrial genome of *H. erinaceus* presented here provides valuable genomic information for further species identification and phylogenetic study of Hericiaceae.

## Supplementary Material

Supplemental Material

## Data Availability

The genome sequence data supporting the findings of this study are available in the NCBI GenBank (https://www.ncbi.nlm.nih.gov/) under accession no. ON527788. The associated BioProject, SRA, and Bio-Sample numbers were PRJNA869545, SRR21047616, and SAMN30309510, respectively.
