# Supplementary material for: The complete mitochondrial genome of Hericium erinaceus (Bull.:Fr.) Pers., 1797 (Russulales, Basidiomycota): an edible and medicinal fungus
Source: Mitochondrial DNA B Resour. 2024 Mar 19;9(3):357–60. doi: 10.1080/23802359.2024.2324923 (PMC10956905; doi:10.1080/23802359.2024.2324923)
Supplement: Supplemental Material [file TMDN_A_2324923_SM6607.pdf]

Mitochondrial DNA Part B: Resources
Date submitted: 28 July 2023

Date revised: 25 September 2023

Running head:

The Complete Mitochondrial Genome of *Hericium erinaceus* (Bull.:Fr.) Pers., 1797 (Russulales, Basidiomycota): An edible and medicinal fungus

Mei Wang[1,3+], Fei Xu[2+], Xiaomei Hu[1], Jianfei Chen[3], Xiaoya Song[4] &Tingting Song[3*]
[1]*Collage of Life Science, Northeast Agricultural University, Harbin, Heilongjiang, 150000, People's Republic of China*

[2] *State Key Laboratory for Managing Biotic and Chemical Threats to the Quality and Safety of Agro-products, Zhejiang Academy of Agricultural Sciences, Hangzhou, Zhejiang, 310021, People's Republic of China*

[3]*Institute of Horticulture, Zhejiang Academy of Agricultural Sciences, Hangzhou, Zhejiang, 310021, People's Republic of China*

[4]*Lishui Academy of Agricultural and Forestry Sciences, Lishui, Zhejiang 323000, People's Republic of China*

[+] The authors contributed equally to this paper.

*Corresponding author: Tingting Song, Institute of Horticulture, Zhejiang Academy of Agricultural Sciences, Hangzhou, Zhejiang, 310021, People's Republic of China; E-mail: Song_tt@sina.com; Tel. & Fax: 86-571-86404017

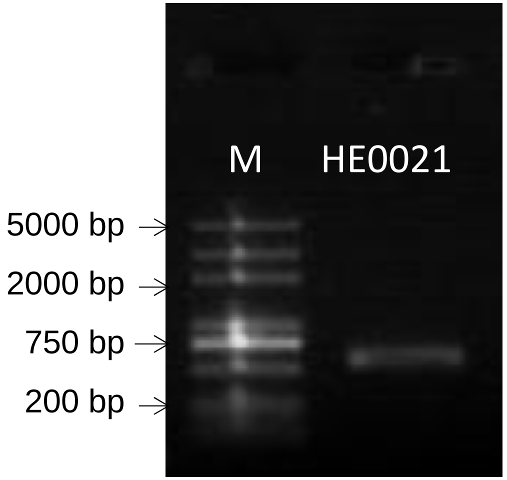

The sequencing result:
>HE0021
tatgcttaagttcagcgggtagtcccgcctgattcgaggtcaagttgagatgaaagttgtccgaagacggttagaag
cagaccaacgcaaggctacccgcggcgtagataattatcacaccaagggcaaaccctacaaaagagatccac
tcatgcattcaagaggagccgacttcccgagggagcaccggcaagacctccaagtccaagcccagccctctcat
aacaagaggattgagttgagaatttcacgacactcgaacaggcgtgcccctcggaataccaagggcgcaagg
tgcgttcaaagattcgatgattcactgaattctgcaattcacattacttatcgcatttcgctgcgttcttcatcgatgcgag
agccaagagatccgttgttgaaagttgtataagatgcgtttatagcattatgacattctgttacatacataagagtttatg
aaaaacgcaagcccatcgaccgcaaagccgacggacccacgcaagggtgcacaggtgtaagatggatgag
attggagcgtgcacatgcctgggtttcaggccagcaacaactcctttcaaattcattaatgatccttccgca

Figure S1. Ampplification of ITS region of rDNA was carried out using universal primers ITS1 and ITS4 (Left); and the partial sequence of ITS sequence(601 bp) in HE0021 stain(Right).

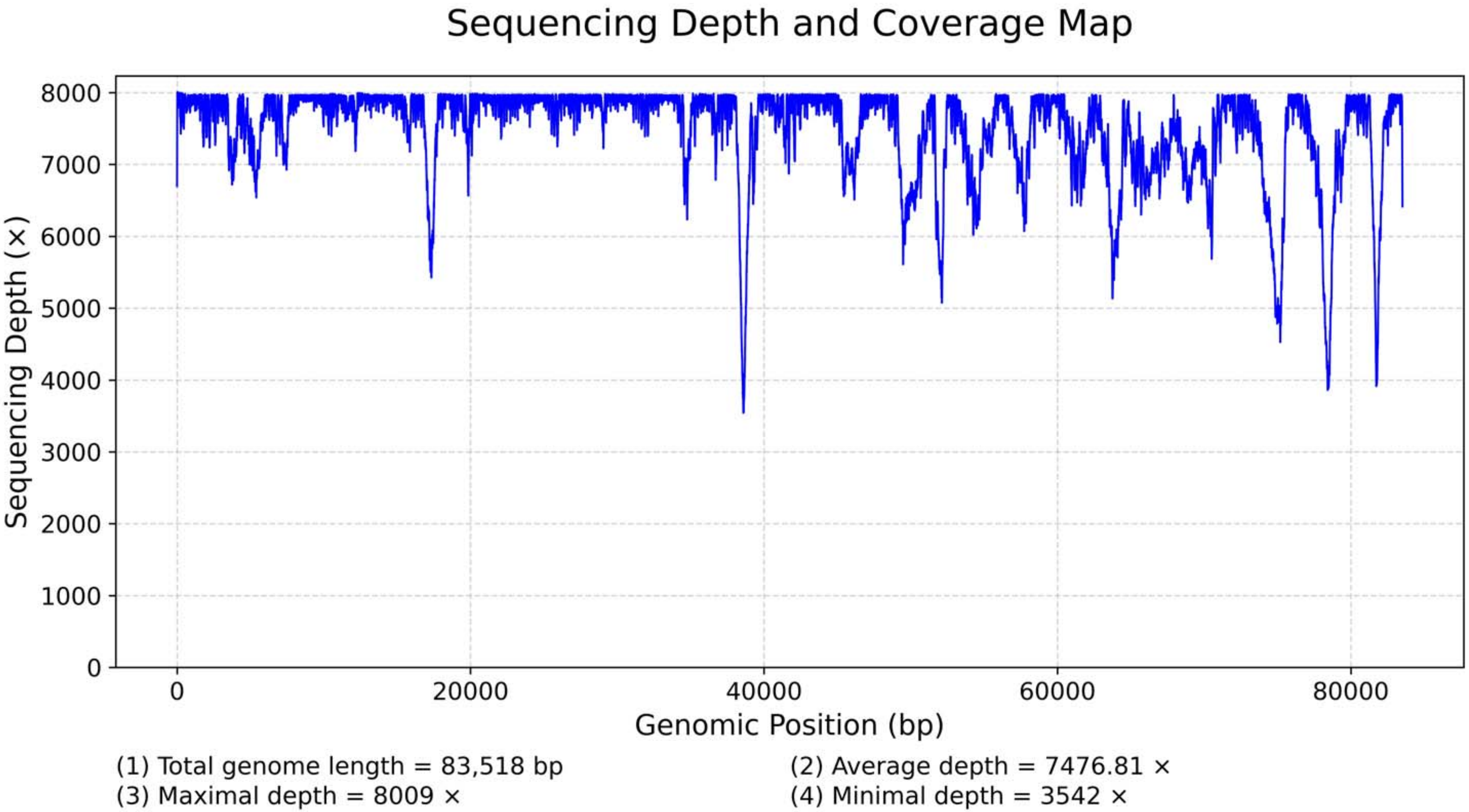

Figure S2. Sequencing depth and coverage map for mitochondrial genome of *Hericium erinaceus* HE0021 (The X-axis indicates the locationof bases in the genome; Y-axis indicates the coverage depth).
